# Forces on and in the cell walls of living plants

**DOI:** 10.1093/plphys/kiad387

**Published:** 2023-07-04

**Authors:** Michael C Jarvis

**Affiliations:** College of Science and Engineering, University of Glasgow, Glasgow G12 8QQ, Scotland, UK

## Abstract

Environmental influences and differential growth subject plants to mechanical forces. Forces on the whole plant resolve into tensile forces on its primary cell walls and both tensile and compression forces on the secondary cell wall layers of woody tissues. Forces on cell walls are further resolved into forces on cellulose microfibrils and the noncellulosic polymers between them. Many external forces on plants oscillate, with time constants that vary from seconds to milliseconds. Sound waves are a high-frequency example. Forces on the cell wall lead to responses that direct the oriented deposition of cellulose microfibrils and the patterned expansion of the cell wall, leading to complex cell and tissue morphology.

Recent experiments have established many of the details of which cell wall polymers associate with one another in both primary and secondary cell walls, but questions remain about which of the interconnections are load bearing, especially in primary cell walls. Direct cellulose–cellulose interactions appear to have a more important mechanical role than was previously thought, and some of the noncellulosic polymers may have a role in keeping microfibrils apart rather than cross-linking them as formerly envisaged.

## Introduction

The walls of plant cells have to withstand both external forces imposed by the plant's environment and internal forces driving, controlling, and consequent upon growth. Recent developments in our understanding of these forces are reviewed here. There have, however, been seminal recent advances in our understanding of forces on plants, how these forces are carried by cell walls of different types, and how force is transmitted through the network of polymers that comprise the cell wall. There is scope to draw these advances together. For example, there has not recently been much comparison of primary and secondary cell walls (Cosgrove and Jarvis 2012). Insights on how cell walls respond to force will underpin future concepts of plant form, mechanics, and morphogenesis.

### Strategies for strength in plants: primary and secondary cell walls

The size, shape, and rigidity of a small plant are maintained by flexible primary cell walls, inflated, and thus held in tension by the turgor pressure within the cell (Coen and Cosgrove 2023). There may be additional tensioning by swelling forces linked to water activity within the wall (Jarvis 1992; Zhang and Zhang 2020). The tensile capability of the primary cell wall is—somehow—combined with the capability to permit, control, and direct growth (Cosgrove 2022).

The rigidity that comes from tensioned primary cell walls is sufficient for herbaceous plant stems, leaf laminae, and fine roots to withstand the forces that the environment imposes. That strategy is an economical way to invest fixed carbon: primary cell walls are thin, and about 90% of their mass is water. However, there are forces that the primary wall cannot withstand alone. Even in a small plant, the xylem elements have to withstand negative pressure during transpiration, and their primary walls are reinforced with secondary thickenings, often initially annular or helical so that they can prevent radial collapse (Turner and Somerville 1997) while permitting axial growth (Karam 2005).

In a plant stem that grows to more than about 1 m, turgor cannot cope with the compressive stresses associated with bending and buckling (Cosgrove and Jarvis 2012). Xylem cells in trees and in tall herbaceous plants like sunflowers acquire rigid, woody secondary walls (Karam 2005) that carry both compression and tensile loads (Cosgrove and Jarvis 2012). Stiffness as well as strength is needed to prevent failure by buckling in compression. In young trees and cereal straw, the woody stem is flexible, allowing it to bend with the wind (Gardiner et al. 2016; Jaff and Jarvis 2021). When the wind load is shed by bending, however, the weight of the upper part of the plant adds to the bending force. Grain-laden cereal straw or snow-laden young trees can be trapped and bent to the ground by gravity (Jaff and Jarvis 2021). Tall trees with large mass have to rely on stiffness to limit bending and the gravitational load that accompanies it (Altaner and Jarvis 2008). Stiffness increases with the fourth power of the stem diameter if the properties of the wood are constant, but in addition, stiffer wood, with more axial cellulose orientation, is laid down in the outermost, last-formed region of the stem, which provides most of the resistance to bending (Altaner and Jarvis 2008). Because trees grow radially as well as upward, a mature tree still has its young self inside.

In angiosperm wood the functions of water conduction and reinforcement against bending are divided between vessels and sclerenchyma, whereas in gymnosperm wood these functions are combined in tracheids (Koch et al. 2004; Pandey 2021). Both strategies permit trees to reach heights over 100 m, simultaneously limited by water transport and wind resistance (Koch et al. 2004), but the cost in fixed carbon is high: that is why the largest carbon store in the terrestrial biosphere is wood (Schimel 1995).

### External forces on whole plants

It is useful to distinguish between constant and fluctuating (dynamic) forces that plants need to sustain, whichever of the above mechanical strategies they exhibit. Gravity loads on plants, due to their own weight or additional snow loading (Ray and Bret-Harte 2019; Zhang et al. 2021a), are in general approximately constant, as are internal forces associated with turgor or xylem transport; but many external forces oscillate (Ram et al. 2022). Oscillating forces have been invoked as a basis for proprioception in plants (Moulia et al. 2021).

The bending of a plant in the wind leads to both static and oscillating forces in the stem. The static component depends on the mean wind speed and comprises tension on the windward side, compression (additional to gravity) on the opposite side, and shear between. But in addition, the crown of a tree and the outer end of each branch follow circular paths with a timescale of the order of seconds (Gardiner et al. 2016; Tadrist et al. 2018), and the forces at any point in the stem therefore oscillate. Herbaceous plants such as cereals behave similarly: their natural, resonant frequency is higher, but low-frequency motions are also present due to turbulence in the airflow (Gardiner et al. 2016), as can be seen when wind ripples across a field. There are higher-frequency (10–100 Hz) oscillating stresses when individual leaves flutter in the wind (Tadrist et al. 2018).

In buzz pollination, the pollinating bee grasps and vibrates the stamen, often around its resonant frequency (0.1–1 kHz) (Garcia Brito et al. 2020). Higher-frequency oscillating stresses may be described as sound. Sound waves travelling within plants can result from herbivory (Dou et al. 2021), xylem cavitation (Venturas et al. 2017), branches breaking under wind or snow load (Zhang et al. 2021a), snap freezing in the xylem (Lintunen 20), or drumming by woodpeckers (Schuppe et al. 2021). Paths of sound transmission in plants are complex but follow cellulose fibers due to their elastic stiffness; the axial speed of sound in wood at kHz to MHz frequencies is used to measure its engineering stiffness (Lachenbruch et al. 2010) and its suitability for making musical instruments (Su et al. 2021), both of which characteristics depend on cellulose orientation (Lachenbruch et al. 2010).

Plants also respond to sound waves of external origin (Gagliano et al. 2017; Liu et al. 2017; Bhandawat and Jayaswall 2022; Del Stabile et al. 2022). It is probable that external sounds can be transduced into internal vibrations wherever they impact, but small plant structures such as trichomes may be adapted to resonate with specific frequencies of external sound transduction and communicate these vibrational signals through their points of attachment (Liu et al. 2017; Yin et al. 2021).

**ADVANCES BOX**

- Many of the external forces on plants oscillate, with frequencies from Hz to KHz. This includes external sound waves, to which plants are now known to respond.
- Feedback responses to growth-driven forces on primary cell walls contribute to plant morphogenesis.
- The associations between polymers in primary cell walls differ from what was previously thought. In particular, pectins are closely associated with cellulose microfibrils
- Direct cellulose–cellulose contacts, local in primary cell walls but extensive in wood, transmit shear stresses between microfibrils.

### Force transmission to cell walls

In a spherical suspension-cultured cell, the wall is under uniform tension, but the cells of living plants are attached to one another and take many other shapes. The uniform pressure of turgor therefore leads to highly non-uniform patterns of forces on the cell walls. The turgor forces on the walls separating adjacent cells are approximately in balance, but the outer wall of an epidermal cell needs to constrain the pressure exerted by that cell and cells inside (Coen and Cosgrove 2023). The unusual shapes of some epidermal cells, like jigsaw pieces, are thought to be an adaptation to these forces on their outer walls (Zhang et al. 2021b).

For more than half a century it has been suggested that the geometric interplay of local forces and locally anisotropic cell wall mechanics lies behind much of the complexity of plant morphogenesis (Green 1964). Evidence elaborating this view is now taking shape, with leaf laminae (Zhao et al. 2020), shoot apices (Sampathkumar et al. 2019; Peaucelle 2020; Moulia et al. 2022), and pollen tubes (Cameron and Geitmann 2018) as examples and with feedback from local force to cellulose orientation as a key component (Jonsson et al. 2022). The forces concerned are often constant, but nonconstant endogenous forces are associated with the slowly oscillating growth of pollen tubes (Pietruszka et al. 2018), opening and closing of stomata (Auler et al. 2022), and deformations around pulvini during rapid plant movements (Mano and Hasebe 2021).

At the single-cell level, tensile stresses are greatest across the narrowest part of a jigsaw-shaped epidermal cell, and the microfibril alignment there corresponds to the stress direction (Altartouri et al. 2019; Sampathkumar and Meyerowitz 2021). At cell corners, turgor is resolved into forces pulling each cell away from its neighbors (Jarvis 1998) because the optimum shape for a flexible, inflated cell wall is spherical. Recent developments in this rapidly expanding field of mechanically modulated morphogenesis will not be detailed here because good, recent reviews are available (Duy-Chi et al. 2021; Codjoe et al. 2022; Coen and Cosgrove 2023).

Primary cell walls can adapt to changing stresses during growth, but wood cells are dead and cannot adapt. Thus as a branch grows in length and weight (Ray and Bret-Harte 2019), an asymmetric distribution of force between top and bottom must be introduced to prevent downward bending (Gril et al. 2017). This force is supplied by the synthesis of reaction wood: tension wood along the top of an angiosperm branch, compression wood on the underside of a conifer branch (Gril et al. 2017). Trees induced to lean form reaction wood in the main trunk. A single stormy day leaves a detectable file of compression wood cells on the side of a spruce tree away from the wind (Altaner et al. 2007).

The forces generated by reaction wood in trees are large and permanent. There is not yet a consensus on how the tensile forces in tension wood and the expansion forces in compression wood are generated by interaction of the cell wall polymers, but competing theories were critically reviewed by Almeras and Clair (2016). More recent research has highlighted anatomical variation in tension wood, with a contribution from fibers at the vascular cambium (Ghislain et al. 2019). Even the deposition of ordinary wood generates growth stress (Thibaut and Gril 2021), detectable by elongation of the crystallographic unit cell of cellulose (Almeras and Clair 2016). Some Eucalyptus species are disfavored for sawn timber because their logs explosively disintegrate when the internal stresses are released in the sawmill (Guo et al. 2019).

### Forces within cell walls

Forces on plant cell walls resolve into forces on their polymer components, particularly forces on cellulose microfibrils (Coen and Cosgrove 2023). To understand how cell walls resist force, we need to know their structure and how it redistributes stress among the diverse cell wall polymers. There is growing experimental knowledge about force distribution among wood polymers. The stretching and reorientation of cellulose microfibrils can be measured by X-ray diffraction under stress (Keckes et al. 2003; Thomas et al. 2021), and partial information on the stretching and reorientation of noncellulosic wood polymers can be extracted from the polarized vibrational spectra of wood under either static or dynamic stress (Salmen et al. 2016; Thomas et al. 2021; Salmen 2022).

In wood the secondary cell walls typically have 3 layers: S1, S2, and S3. The middle S2 layer dominates the mass of the wall and has a microfibril angle that varies from near zero to 30° or more, the lower figures being for stiff mature wood (Altaner and Jarvis 2008). The higher microfibril angles are found in more pliant juvenile wood and compression wood in conifers (Keckes et al. 2003). The way in which the S2 microfibrils are wound around the cell would tend to contract its diameter under axial tension, but this tendency is resisted by the outer S1 and inner S3 layers, where the microfibrils are circumferentially oriented. If the wood cells are sliced lengthwise, their tensile stiffness is reduced because the hoop restraint of the S1 and S3 wall layers is destroyed (Guo et al. 2019).

Cells that form part of the structure of wood cannot twist. A consequence is that in the dominant S2 layer, microfibril reorientation, and shear are balanced (Keckes et al. 2003). Stretching of the microfibrils becomes prominent in the stiffest wood, where the microfibril angle is small (<10°). However, the extent of elongation varies between microfibrils from zero to over twice the mean (Thomas et al. 2021). This variation implies that shear between loaded and unloaded microfibril segments is extensive even when they are approximately parallel. Interfibrillar shear stresses are carried at least partly by direct cellulose–cellulose contacts, although hemicelluloses and probably lignin domains also participate (Jarvis 2023).

Depending on the microfibril angle, interfibrillar shear stresses also result from deformation across the grain (Keckes et al. 2003). At large compressive strains, cell walls buckle, leading to complex local stresses on microfibrils that are difficult to analyze but probably involve both bending and shear (Sun et al. 2021). Tension on microfibrils is predominantly reversible, whereas shear can be irreversible above a threshold stress (Keckes et al. 2003; Zhang et al. 2021b), with likely consequences for the irreversible, energy-absorbing deformation of wood, which does not permit growth but provides resilience against shock and oscillating forces (Ray and Bret-Harte 2019).

Experimental techniques used for wood (Burgert and Keplinger 2013) have been less informative for primary cell walls. Atomic force microscopy and other imaging methods have been helpful (Zhang et al. 2017; Haas et al. 2020), but we know less than we should about how the primary cell wall deforms internally under stress or during growth (Cosgrove 2022). In each lamella of a typical primary cell wall, the microfibrils are to some extent bundled and are oriented at a large angle to the cell axis; but the bundling does not extend between successive lamellae (Zhang et al. 2017), and the orientation often sharply differs from one lamella to the next (a crossed lamellate structure) (Cosgrove 2022), in contrast to the microfibrils of the S2 layer of the wood cell wall.

The microfibrils in the primary cell wall are too far off-axis to show measurable stretching under external tension, for example, by diffraction methods. However, in each lamella of a primary wall under external tension, reorientation (Zhang et al. 2017) of bundled cellulose microfibrils can be observed and is presumably balanced by shear as in the S2 layer of wood cell walls (Keckes et al. 2003). Axial elongation is accompanied by lateral contraction as would be expected for a trellis arrangement of microfibrils (Zhang et al. 2017).

This rearrangement of microfibril geometry under tension is apparently not what happens during the elongation growth of living cells. When the primary wall elongates in tension under the influence of wall-loosening enzymes, no reorientation of microfibrils occurs (Marga et al. 2005; Zhang et al. 2017) and the elongation does not lead to lateral contraction (Zhang et al. 2017). Microfibril spacing appears to increase (Marga et al. 2005). It is proposed in these circumstances, more closely (but not fully) simulating turgor-driven growth, the attachment points between microfibrils are disrupted (Cosgrove 2022), unloading the microfibrils like cutting the pivots in a trellis.

Coarse-grained modelling has illuminated how primary cell walls may resist tension (Zhang et al. 2021b). In these models, microfibrils are differentially stressed according to their alignment with the stress on the cell wall. Much of the stress transfer between microfibrils is currently thought to be carried by direct microfibril–microfibril interactions rather than by cross-linking polymers (Zhang et al. 2017), although the arrangements of noncellulosic polymers between the microfibrils, and therefore how these polymers are loaded to transfer shear stress, are not yet understood in enough detail to give a secure functional underpinning to the models (Haas et al. 2021; Zhang et al. 2021b). The signatures of the noncellulosic polysaccharides are rather difficult to disentangle in the polarized vibrational spectra (MacKinnon et al. 2006), which would otherwise give useful information on the relative loading of the different chain types. It is now known that anionic pectic domains are closely associated with microfibrils (Wang et al. 2015; Phyo et al. 2019), but whether these lead to microfibril cohesion, microfibril repulsion, or phase separation and how the more flexible, neutral pectic chain segments are accommodated remains uncertain (MacDougall et al. 1997; Haas et al. 2021; Kirui et al. 2021). Xyloglucans seem to be only locally associated with the microfibrils (Cosgrove 2022). Direct, load-bearing microfibril–microfibril contacts certainly exist (bundling) (Cosgrove 2022), but their abundance and locations are not wholly clear. How extended these contacts are, and hence how concentrated the shear forces are between them, depends on the angle between the microfibrils at the point where they touch (Zhang et al. 2017) and perhaps on the surrounding noncellulosic chains (Wang et al. 2013; Haas et al. 2021). At such microfibril–microfibril contacts, local shear between microfibrils will give rise to local shear within them. Shear within microfibrils occurs also when they bend, is located preferentially between sheets of chains, and may be partially irreversible (Jarvis 2023).

In summary, recent experiments on the tensile cohesion of primary cell walls have brought cellulose–cellulose contacts into focus, but the details of how these contacts are modulated and control the different between tension-mediated elongation and growth remain uncertain.

Cellulose is known to be a piezoelectric material, coupling electricity to force. It might then be speculated that electric fields generated by cellulose contribute to the sensing of external and endogenous forces. However, it transpires that electric fields generated in that way are dissipated too rapidly by conduction for any cellular response to be plausible. The reasoning is explained in Box 1, as a cautionary tale for anyone tempted to think in a similar direction.

Box 1. **Forces on cellulose do not lead to sustained piezoelectric signals in vivo.**

The time constant for dissipation of piezoelectric charge within the cell wall depends on the ratio of the conductance k of the cell wall to the initial charge Q. The relationship of Q (Coulombs) to voltage V is given by the standard definition of capacitance:

$$Q\;=\;V\varepsilon_{r}\varepsilon_{0}.A/d,$$

where ɛ_r_ is the dielectric constant of the cell wall, ɛ_0_ is the vacuum permittivity, d is the length of the cell wall domain, and A is its cross-sectional area.

The initial current I (Coulombs/second) that flows under the same voltage V is given by Ohm's Law:

I=V.kA/d,

where k is the electric conductance of the cell wall. The ratio I/Q = k/ɛ_r_ɛ_0_ since VA/d cancels (thus the rate of dissipation is independent of the dimensions of the system).

Quantitative values of k and ɛ_r_ɛ_0_ are not accessible for primary cell walls but data for wood have been published. A consensus value of k for wet wood is 0.01 S/m (Vermaas 1974), up to 0.1 S/m for thin woody branches. From the ionic composition of apoplastic fluid from *Vigna* hypocotyls (Goldberg et al. 1996), k for primary cell walls is probably also about 0.1 S/m. For wet wood, ɛ_r_ɛ_0_ is about 10^−10^ F/m (Vermaas 1974), so the ratio I/Q = k/ɛ_r_ɛ_0_ is large. This is not the case for dry wood because its conductivity is only about 10^−13^ S/m, increasing exponentially with moisture, whereas the variation of dielectric constant with moisture content is relatively small (Vermaas 1974). So piezoelectric phenomena can be observed in dry wood but are not likely to be observable, nor significant for sensing, in living plant materials.

### Outlook

Given the newly improved understanding of microfibril–microfibril association (Zhang et al. 2021b; Cosgrove 2022) and of which noncellulosic polymer chains are spatially associated with microfibrils (Wang et al. 2015; Terrett et al. 2019; Kirui et al. 2021; Salmen 2022), it should be possible to clarify in more detail how shear forces are transmitted between the microfibrils of both primary and secondary cell walls. Noncellulosic polymers may prove to be as important in keeping microfibril surfaces from cohering as in cross-linking microfibrils. These issues may prove to be central to how plant cells grow and how plant form emerges, as well as to the toughness of wood.

**UNANSWERED QUESTIONS**

Many broad questions about the functioning of cell walls under stress remain unanswered or are not adequately answered. The following is a small selection of specific questions with broader implications.

- Which microfibril faces are preferentially involved in direct interactions with other microfibrils?
- How do microfibril–microfibril interactions depend on the angle between the interacting microfibrils, for example, at the interfaces between primary wall lamellae?
- Why do anionic pectic chains associate with primary wall cellulose, and are they firmly enough associated to disperse microfibrils electrostatically within or between lamellae?
- Where exactly is the lignin in the wood cell wall, and what forces does it carry?

## Conclusions

Primary and secondary cell walls are constructed on similar principles from analogous structural elements. There are parallels in how forces are transmitted between cellulose microfibrils in each case, but the outcomes are different: tensile strength combined with modulated growth in the primary cell wall, tensile and compressive strength combined with modulated stiffness in the secondary cell wall. Sharing concepts and experimental techniques across these boundaries will be helpful in unravelling how all cell walls work.

## References

[kiad387-B1] Almeras T , Clair B. Critical review on the mechanisms of maturation stress generation in trees. J R Soc Interface. 2016:13(122):20160550. 10.1098/rsif.2016.0550 PMC5046956 27605169

[kiad387-B2] Altaner C , Hapca AI, Knox JP, Jarvis MC. Detection of beta-1–4-galactan in compression wood of Sitka spruce *Picea sitchensis* (Bong.) Carriere by immunofluorescence. Holzforschung. 2007:61(3):311–316. 10.1515/hf.2007.049

[kiad387-B3] Altaner CM , Jarvis MC. Modelling polymer interactions of the ‘molecular Velcro’ type in wood under mechanical stress. J Theor Biol. 2008:253(3):434–445. 10.1016/j.jtbi.2008.03.010 18485371

[kiad387-B4] Altartouri B , Bidhendi AJ, Tani T, Suzuki J, Conrad C, Chebli Y, Liu N, Karunakaran C, Scarcelli G, Geitmann A. Pectin chemistry and cellulose crystallinity govern pavement cell morphogenesis in a multi-step mechanism. Plant Physiol. 2019:181(1):127–141. 10.1104/pp.19.00303 31363005 PMC6716242

[kiad387-B5] Auler PA , Freire FBS, Lima VF, Daloso DM. On the role of guard cells in sensing environmental signals and memorising stress periods. Theor Exp Plant Physiol. 2022:34(3):277–299. 10.1007/s40626-022-00250-4

[kiad387-B6] Bhandawat A , Jayaswall K. Biological relevance of sound in plants. Environ Exp Bot. 2022:200:104919. 10.1016/j.envexpbot.2022.104919

[kiad387-B7] Burgert I , Keplinger T. Plant micro- and nanomechanics: experimental techniques for plant cell-wall analysis. J Exp Bot. 2013:64(15):4635–4649. 10.1093/jxb/ert255 24064925

[kiad387-B8] Cameron C , Geitmann A. Cell mechanics of pollen tube growth. Curr Opin Genet Dev. 2018:51:11–17. 10.1016/j.gde.2018.03.008 29602058

[kiad387-B9] Codjoe JM , Miller K, Haswell ES. Plant cell mechanobiology: greater than the sum of its parts. Plant Cell. 2022:34(1):129–145. 10.1093/plcell/koab230 34524447 PMC8773992

[kiad387-B10] Coen E , Cosgrove DJ. The mechanics of plant morphogenesis. Science (New York). 2023:379(6631):eade8055. 10.1126/science.ade8055 36730409

[kiad387-B11] Cosgrove DJ . Building an extensible cell wall. Plant Physiol. 2022:189(3):1246–1277. 10.1093/plphys/kiac184 35460252 PMC9237729

[kiad387-B12] Cosgrove DJ , Jarvis MC. Comparative structure and biomechanics of plant primary and secondary cell walls. Front Plant Sci. 2012:3:204. 10.3389/fpls.2012.00204 22936943 PMC3424969

[kiad387-B13] Del Stabile F , Marsili V, Forti L, Arru L. Is there a role for sound in plants? Plants-Basel. 2022:11(18):2391. 10.3390/plants11182391 36145791 PMC9503271

[kiad387-B14] Dou C , Li M, Luo T, Deng T, Zhu D. Characteristics of acoustic emission signal of *Coscinesthes salicis* larvae in poplar tree segments. J Northeast For Univ. 2021:49(11):95–99.

[kiad387-B15] Duy-Chi T , Alonso-Serra J, Asaoka M, Colin L, Cortes M, Malivert A, Takatani S, Zhao F, Traas J, Trehin C, et al How mechanical forces shape plant organs. Curr Biol. 2021:31(3):R143–R159. 10.1016/j.cub.2020.12.001 33561417

[kiad387-B16] Gagliano M , Grimonprez M, Depczynski M, Renton M. Tuned in: plant roots use sound to locate water. Oecologia. 2017:184(1):151–160. 10.1007/s00442-017-3862-z 28382479

[kiad387-B17] Garcia Brito VL , Pereira Nunes CE, Resende CR, Montealegre-Zapata F, Vallejo-Marin M. Biomechanical properties of a buzz-pollinated flower. R Soc Open Sci. 2020:7(9):201010. 10.1098/rsos.201010 33047057 PMC7540744

[kiad387-B18] Gardiner B , Berry P, Moulia B. Review: wind impacts on plant growth, mechanics and damage. Plant Sci. 2016:245:94–118. 10.1016/j.plantsci.2016.01.006 26940495

[kiad387-B19] Ghislain B , Almeras T, Prunier J, Clair B. Contributions of bark and tension wood and role of the G-layer lignification in the gravitropic movements of 21 tropical tree species. Ann For Sci. 2019:76(4):1–13. 10.1007/s13595-019-0899-7

[kiad387-B20] Goldberg R , Morvan C, Jauneau A, Jarvis MC. Methyl-esterification, de-esterification and gelation of pectins in the primary cell wall. In: International Symposium on Pectins and Pectinases. Wageningen,Netherlands; 1996. p. 151–172. 10.1016/s0921-0423(96)80253-x

[kiad387-B21] Green PB . Cell walls and the geometry of plant growth. Brookhaven Symp Biol. 1964:16:203–217. 14160257

[kiad387-B22] Gril J , Jullien D, Bardet S, Yamamoto H. Tree growth stress and related problems. J Wood Sci. 2017:63(5):411–432. 10.1007/s10086-017-1639-y

[kiad387-B23] Guo F , Cramer M, Altaner CM. Evaluation of near infrared spectroscopy to non-destructively measure growth strain in trees. Cellulose. 2019:26(13–14):7663–7673. 10.1007/s10570-019-02627-2

[kiad387-B24] Haas KT , Riviere M, Wightman R, Peaucelle A. Multitarget immunohistochemistry for confocal and super-resolution imaging of plant cell wall polysaccharides. Bio Protoc. 2020:10(19):e3783. 10.21769/BioProtoc.3783 PMC7842508 33659438

[kiad387-B25] Haas KT , Wightman R, Peaucelle A, Hofte H. The role of pectin phase separation in plant cell wall assembly and growth. Cell surface (Amsterdam, Netherlands). 2021:7:100054–100054. 10.1016/j.tcsw.2021.100054 34141960 PMC8185244

[kiad387-B26] Jaff D , Jarvis MC. Straw bending and lodging in landrace barley: finite element modelling. Sarhad J Agric. 2021:37(2):555–563. 10.17582/journal.sja/2021/37.2.555.563

[kiad387-B27] Jarvis MC . Control of thickness of collenchyma cell walls by pectins. Planta. 1992:187(2):218–220. 10.1007/BF00201941 24178046

[kiad387-B28] Jarvis MC . Intercellular separation forces generated by intracellular pressure. Plant Cell Environ. 1998:21(12):1307–1310. 10.1046/j.1365-3040.1998.00363.x

[kiad387-B29] Jarvis MC . Hydrogen bonding and other non-covalent interactions at the surfaces of cellulose microfibrils. Cellulose. 2023;30:667–687. 10.1007/s10570-022-04954-3

[kiad387-B30] Jonsson K , Hamant O, Bhalerao RP. Plant cell walls as mechanical signaling hubs for morphogenesis. Curr Biol. 2022:32(7):R334–R340. 10.1016/j.cub.2022.02.036 35413265

[kiad387-B31] Karam GN . Biomechanical model of the xylem vessels in vascular plants. Ann Bot. 2005:95(7):1179–1186. 10.1093/aob/mci130 15802309 PMC4246902

[kiad387-B32] Keckes J , Burgert I, Fruhmann K, Muller M, Kolln K, Hamilton M, Burghammer M, Roth SV, Stanzl-Tschegg S, Fratzl P. Cell-wall recovery after irreversible deformation of wood. Nat Mater. 2003:2(12):810–814. 10.1038/nmat1019 14625541

[kiad387-B33] Kirui A , Du J, Zhao W, Barnes W, Kang X, Anderson CT, Xiao C, Wang T. A pectin methyltransferase modulates polysaccharide dynamics and interactions in Arabidopsis primary cell walls: evidence from solid-state NMR. Carbohydr Polym. 2021:270:118370. 10.1016/j.carbpol.2021.118370 34364615

[kiad387-B34] Koch GW , Sillett SC, Jennings GM, Davis SD. The limits to tree height. Nature. 2004:428(6985):851–854. 10.1038/nature02417 15103376

[kiad387-B35] Lachenbruch B , Johnson GR, Downes GM, Evans R. Relationships of density, microfibril angle, and sound velocity with stiffness and strength in mature wood of Douglas-fir. Can J For Res. 2010:40(1):55–64. 10.1139/x09-174

[kiad387-B36] Liu S , Jiao J, Lu TJ, Xu F, Pickard BG, Genin GM. Arabidopsis leaf trichomes as acoustic antennae. Biophys J. 2017:113(9):2068–2076. 10.1016/j.bpj.2017.07.035 29117529 PMC5685652

[kiad387-B37] MacDougall AJ , Rigby NM, Ring SG. Phase separation of plant cell wall polysaccharides and its implications for cell wall assembly. Plant Physiol. 1997:114(1):353–362. 10.1104/pp.114.1.353 12223708 PMC158311

[kiad387-B38] MacKinnon IM , Šturcová A, Sugimoto-Shirasu K, His I, McCann MC, Jarvis MC. Cell-wall structure and anisotropy in *procuste*, a cellulose synthase mutant of *Arabidopsis thaliana*. Planta. 2006:224(2):438–448. 10.1007/s00425-005-0208-6 16404578

[kiad387-B39] Mano H , Hasebe M. Rapid movements in plants. J Plant Res. 2021:134(1):3–17. 10.1007/s10265-020-01243-7 33415544 PMC7817606

[kiad387-B40] Marga F , Grandbois M, Cosgrove DJ, Baskin TI. Cell wall extension results in the coordinate separation of parallel microfibrils: evidence from scanning electron microscopy and atomic force microscopy. Plant J. 2005:43(2):181–190. 10.1111/j.1365-313X.2005.02447.x 15998305

[kiad387-B41] Moulia B , Badel E, Bastien R, Duchemin L, Eloy C. The shaping of plant axes and crowns through tropisms and elasticity: an example of morphogenetic plasticity beyond the shoot apical meristem. New Phytol. 2022:233(6):2354–2379. 10.1111/nph.17913 34890051

[kiad387-B42] Moulia B , Douady S, Hamant O. Fluctuations shape plants through proprioception. Science. 2021:372(6540):359. 10.1126/science.abc6868 33888615

[kiad387-B43] Pandey S . Climatic influence on tree wood anatomy: a review. J Wood Sci. 2021:67(1):1–7. 10.1186/s10086-021-01956-w

[kiad387-B44] Peaucelle A . Exploring the relation between apical growth, organ formation and cell wall mechanics across land plant species. C R Mecanique. 2020:348(6–7):685–692. 10.5802/crmeca.28

[kiad387-B45] Phyo P , Gu Y, Hong M. Impact of acidic pH on plant cell wall polysaccharide structure and dynamics: insights into the mechanism of acid growth in plants from solid-state NMR. Cellulose. 2019:26(1):291–304. 10.1007/s10570-018-2094-7

[kiad387-B46] Pietruszka M , Olszewska M, Machura L, Rowinski E. Single measurement detection of individual cell ionic oscillations using an n-type semiconductor-electrolyte interface. Sci Rep. 2018:8(1):7875. 10.1038/s41598-018-26015-1 29777196 PMC5959918

[kiad387-B47] Ram F , Garemark J, Li YY, Pettersson T, Berglund LA. Functionalized wood veneers as vibration sensors: exploring wood piezoelectricity and hierarchical structure effects. ACS Nano. 2022:16(10):15805–15813. 10.1021/acsnano.2c04668 36067037 PMC9620403

[kiad387-B48] Ray PM , Bret-Harte MS. Elastic and irreversible bending of tree and shrub branches under cantilever loads. Front Plant Sci. 2019:10:59. 10.3389/fpls.2019.00059 30804957 PMC6370663

[kiad387-B49] Salmen L . On the organization of hemicelluloses in the wood cell wall. Cellulose. 2022:29(3):1349–1355. 10.1007/s10570-022-04425-9

[kiad387-B50] Salmen L , Stevanic JS, Olsson A-M. Contribution of lignin to the strength properties in wood fibres studied by dynamic FTIR spectroscopy and dynamic mechanical analysis (DMA). Holzforschung. 2016:70(12):1155–1163. 10.1515/hf-2016-0050

[kiad387-B51] Sampathkumar A , Meyerowitz EM. Cellulose synthase complexes-microtubules interaction hinders mechano-response. Nat Plants. 2021:8(9):988–989. 10.1038/s41477-022-01221-y 36028772

[kiad387-B52] Sampathkumar A , Peaucelle A, Fujita M, Schuster C, Persson S, Wasteneys GO, Meyerowitz EM. Primary wall cellulose synthase regulates shoot apical meristem mechanics and growth. Development. 2019:146(10):dev179036. 10.1242/dev.179036 PMC6550022 31076488

[kiad387-B53] Schimel DS . Terrestrial ecosystems and the carbon cycle. Glob Chang Biol. 1995:1(1):77–91. 10.1111/j.1365-2486.1995.tb00008.x 25472464

[kiad387-B54] Schuppe ER , Rutter AR, Roberts TJ, Fuxjager MJ. Evolutionary and biomechanical basis of drumming behavior in woodpeckers. Front Ecol Evol. 2021:9(10):649146. 10.3389/fevo.2021.649146

[kiad387-B55] Su C-K , Chen S-Y, Chung J-H, Li G-C, Brandmair B, Huthwelker T, Fulton JL, Borca CN, Huang S-J, Nagyvary J, et al Materials engineering of violin soundboards by Stradivari and Guarneri. Angew Chem Int Ed. 2021:60(35):19144–19154. 10.1002/anie.202105252 PMC8457145 34062043

[kiad387-B56] Sun J , Guo H, Schadli GN, Tu K, Schar S, Schwarze FWMR, Panzarasa G, Ribera J, Burgert I. Enhanced mechanical energy conversion with selectively decayed wood. Sci Adv. 2021:7(11):eabd9138. 10.1126/sciadv.abd9138 PMC7946366 33692104

[kiad387-B57] Tadrist L , Saudreau M, Hemon P, Amandolese X, Marquier A, Leclercq T, de Langre E. Foliage motion under wind, from leaf flutter to branch buffeting. J R Soc Interface. 2018:15(142):201800. 10.1098/rsif.2018.0010 PMC6000177 29743271

[kiad387-B58] Terrett OM , Lyczakowski JJ, Yu L, Iuga D, Franks WT, Brown SP, Dupree R, Dupree P. Molecular architecture of softwood revealed by solid-state NMR. Nat Commun. 2019:10(1):4978–4978. 10.1038/s41467-019-12979-9 31673042 PMC6823442

[kiad387-B59] Thibaut B , Gril J. Tree growth forces and wood properties. Peer Commun J. 2021:1:e46. 10.24072/pcjournal.48

[kiad387-B60] Thomas LH , Altaner CM, Forsyth VT, Mossou E, Kennedy CJ, Martel A, Jarvis MC. Nanostructural deformation of high-stiffness spruce wood under tension. Sci Rep. 2021:11(1):453. 10.1038/s41598-020-79676-2 33432070 PMC7801420

[kiad387-B61] Turner SR , Somerville CR. Collapsed xylem phenotype of Arabidopsis identifies mutants deficient in cellulose deposition in the secondary cell wall. Plant Cell. 1997:9(5):689–701. 10.1105/tpc.9.5.689 9165747 PMC156949

[kiad387-B62] Venturas MD , Sperry JS, Hacke UG. Plant xylem hydraulics: what we understand, current research, and future challenges. J Integr Plant Biol. 2017:59(6):356–389. 10.1111/jipb.12534 28296168

[kiad387-B63] Vermaas HF . Note on mechanism of electrical conduction in wood. Holzforschung. 1974:28(4):145–148. 10.1515/hfsg.1974.28.4.145

[kiad387-B64] Wang T , Park YB, Caporini MA, Rosay M, Zhong L, Cosgrove DJ, Hong M. Sensitivity-enhanced solid-state NMR detection of expansin's Target in plant cell walls. Proc Natl Acad Sci USA. 2013:110(41):16444–16449. 10.1073/pnas.1316290110 24065828 PMC3799313

[kiad387-B65] Wang T , Park YB, Cosgrove DJ, Hong M. Cellulose-pectin spatial contacts are inherent to never-dried Arabidopsis primary cell walls: evidence from solid-state nuclear magnetic resonance. Plant Physiol. 2015:168(3):871–884. 10.1104/pp.15.00665 26036615 PMC4741345

[kiad387-B66] Yin J , Liu H, Jiao J, Peng X, Pickard BG, Genin GM, Lu TJ, Liu S. Ensembles of the leaf trichomes of *Arabidopsis thaliana* selectively vibrate in the frequency range of its primary insect herbivore. Extreme Mech Lett. 2021:48:101377. 10.1016/j.eml.2021.101377

[kiad387-B69] Zhang D , Zhang B. Pectin drives cell wall morphogenesis without turgor pressure. Trends Plant Sci. 2020:25(8):719–722. 10.1016/j.tplants.2020.05.007 32513584

[kiad387-B70] Zhang M , Zhang Q, Li J, Xu J, Zheng J. Classification of acoustic emission signals in wood damage and fracture process based on empirical mode decomposition, discrete wavelet transform methods, and selected features. J Wood Sci. 2021a:67(1):1–13. 10.1186/s10086-021-01990-8

[kiad387-B67] Zhang T , Vavylonis D, Durachko DM, Cosgrove DJ. Nanoscale movements of cellulose microfibrils in primary cell walls. Nat Plants. 2017:3(5):1–6. 10.1038/nplants.2017.56 PMC5478883 28452988

[kiad387-B68] Zhang Y , Yu J, Wang X, Durachko DM, Zhang S, Cosgrove DJ. Molecular insights into the complex mechanics of plant epidermal cell walls. Science. 2021b:372(6543):706–711. 10.1126/science.abf2824 33986175

[kiad387-B71] Zhao F , Du F, Oliveri H, Zhou LW, Ali O, Chen WQ, Feng SL, Wang QQ, Lu SQ, Long M, et al Microtubule-mediated wall anisotropy contributes to leaf blade flattening. Curr Biol. 2020:30(20):3972–3985. 10.1016/j.cub.2020.07.076 32916107 PMC7575199

